# An Investigation of the Wear on Silicon Surface at High Humidity

**DOI:** 10.3390/ma11061027

**Published:** 2018-06-16

**Authors:** Xiaodong Wang, Jian Guo, Lin Xu, Guanggui Cheng, Linmao Qian

**Affiliations:** 1 Center of Micro/Nano Science and Technology, Jiangsu University, Zhenjiang 212013, China; wangxd@ujs.edu.cn (X.W.); xulin1982@ujs.edu.cn (L.X.); ggcheng@ujs.edu.cn (G.C.); 2 Tribology Research Institute, State Key Laboratory of Traction Power, Southwest Jiaotong University, Chengdu 610031, China; guojian5024@126.com; 3 School of Mechanical Engineering, University of South China, Hengyang 421000, China

**Keywords:** monocrystalline silicon, wear, high humidity, etch

## Abstract

Using an atomic force microscope (AFM), the wear of monocrystalline silicon (covered by a native oxide layer) at high humidity was investigated. The experimental results indicated that tribochemistry played an important role in the wear of the silicon at different relative humidity levels (RH = 60%, 90%). Since the tribochemical reactions were facilitated at 60% RH, the wear of silicon was serious and the friction force was around 1.58 μN under the given conditions. However, the tribochemical reactions were restrained when the wear pair was conducted at high humidity. As a result, the wear of silicon was very slight and the friction force decreased to 0.85 μN at 90% RH. The slight wear of silicon at high humidity was characterized by etching tests. It was demonstrated that the silicon sample surface was partly damaged and the native oxide layer on silicon sample surface had not been totally removed during the wear process. These results may help us optimize the tribological design of dynamic microelectromechanical systems working in humid conditions.

## 1. Introduction

As an excellent structural material, monocrystalline silicon has been widely used in optical devices and microelectromechanical systems (MEMS) [1,2,3,4]. Considering that some MEMS may work in wet air [1,2,3,4,5,6], the wear of silicon in varied humid conditions becomes an important issue. It has been reported that the wear on silicon surface changes with the variation of humidity in normal humid air (RH < 70%) [7,8,9]. However, the wear behaviors and mechanism of silicon at high humidity (RH > 70%) are far less understood [7,10,11].

Due to the difficulty in controlling the environment at a high humid level, a number of previous studies have focused on the investigation of the wear at normal humidity [9,10,12]. Using an AFM, Chung and Kim [12] performed the wear tests of silicon against a silicon AFM tip at RH = 20%, 40%, and 60%. It was indicated that the wear volume of silicon would increase with the increase of RH value. Chen et al. [9] researched the wear of silicon at RH = 10–65%. Based on the results, it was found that the wear of silicon would aggravate at 65% RH. Furthermore, the transmission electron microscope (TEM) results also indicated that the wear of silicon was not dominated by mechanical interactions [9,10]. Only a few papers investigated the wear of silicon at high humidity. For example, Wang et al. [7] studied the wear of silicon at RH = 0–90% by using an improved atomic force microscope. According to the results, the wear behaviors of silicon at 90% RH were different from that at RH < 70%. Even so, the wear mechanism and the damage on silicon surface at high humidity still remained to be revealed.

Since the silicon surface in silicon-based MEMS is always covered by a native oxide layer [12], the wear of monocrystalline silicon against a SiO_2_ tip was performed using an AFM at RH = 60%, 90%. The wear behaviors and the damage on silicon surface after the wear tests will be investigated. The different wear mechanisms of silicon at 60% RH and 90% RH will be proposed. The results of this paper will be helpful to optimize the tribological design of MEMS working in humid conditions.

## 2. Materials and Methods

**Figure 1:**
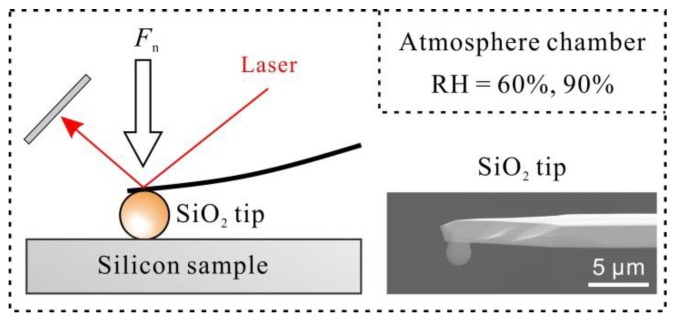
Illustration showing the wear tests of Si/SiO_2_ pair by AFM in a humidity control chamber.

All the wear tests were performed by an AFM (SPI3800N, Seiko, Tokyo, Japan) and humidity was adjusted with an atmosphere chamber, as shown in Figure 1. A p-doped Si(100) wafer (MEMC Electronic Materials, Inc., St. Peters, MO, USA) with a thickness of 0.5 mm was used as the silicon sample and the root–mean–square (RMS) roughness of the sample was characterized as 0.07 nm over a 500 nm × 500 nm area. In order to simulate the dynamic processes of silicon-based MEMS devices, the native oxide layer (SiO_x_), with a thickness of 0.5 nm on silicon sample surface, was not removed by any chemical method [4,5,6].

The wear tests were conducted using spherical SiO_2_ tips with a radius (*R*) of 1 μm (Novascan Technologies, Ames, IA, USA; the SEM image was shown in Figure 1). The spring constant (*k*) of the SiO_2_ tips was calibrated as ~12 N/m. During the wear tests, the applied normal load *F_n_* was set as 3 μN, and the contact pressure *P*_c_ was around 1.2 GPa [13,14,15,16]. In order to get distinct wear results, all the wear tests were carried out by scratching in area. The size of the wear region *A* was set as 1 μm × 1 μm, the number of wear cycles *N* was 2, and the relative speed *v* between the SiO_2_ tip and silicon sample was set as 4 μm/s. Before the wear tests, the SiO_2_ tips were rubbed at different angles in the vacuum condition to ensure the cleanness of the tips. After the wear tests, the silicon samples were immersed in alkaline solution to characterize the damage of silicon sample surface in different humid conditions. The alkaline solution was a mixture of 10 wt % KOH solution and isopropyl alcohol (IPA) (volume ratio = 200:1). All the etching tests were performed at room temperature, set to 23 ± 1 °C. The wear scars after wear tests and etching tests were scanned by a Si_3_N_4_ tip (MLCT, Veeco, Plainview, NY, USA) in vacuum. The radius of the Si_3_N_4_ tip was 20 nm and the nominal normal spring constant was ~0.1 N/m.

## 3. Results

### 3.1. Wear of Silicon at 60% RH and 90% RH

**Figure 2:**
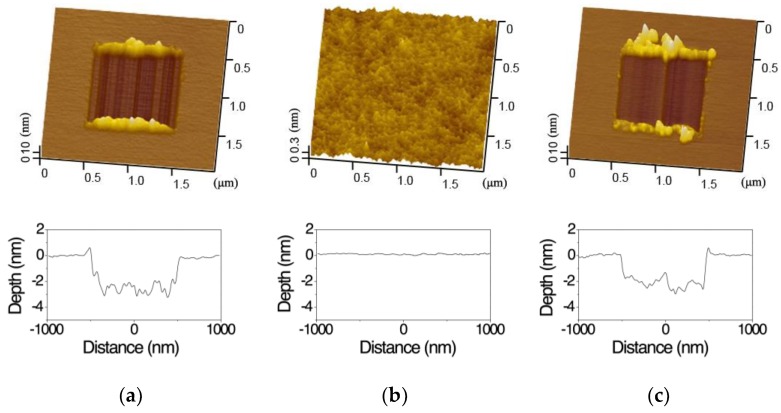
AFM images of wear scars on silicon surface at 60% RH and 90% RH. (**a**) First wear test at 60% RH; (**b**) second wear test at 90% RH; (**c**) third wear test at 60% RH. *F_n_* = 3 μN, *N* = 2, *A* = 1 μm × 1 μm, *v* = 4 μm/s.

In order to locate the wear scars on silicon surface in different humid conditions, a series of scratch marks were prepared before the wear tests. Meanwhile, a repeated wear test of silicon at 60% RH was performed to seek out the wear reason of silicon at 90% RH. The topography and depth of wear scars formed on the silicon surface are shown in Figure 2. The wear of silicon was serious at 60% RH. Wear depth was around 3 nm and a lot of wear debris formed on the both ends of sliding direction, as shown in Figure 2a. However, the wear on the silicon sample surface was restrained and no noticeable wear scar formed when the value of RH increased from 60% to 90%, as shown in Figure 2b. It was also found that the roughness of the wear scar was close to that of the silicon sample surface.

Since the slight wear of silicon at 90% RH might be caused by the wear of SiO_2_ tip, a repeated wear of silicon at 60% RH was carried out after the wear test of silicon at 90% RH. According to the results of Figure 2c, it was demonstrated that the wear of silicon became serious again and the wear depth of the wear scar returned to ~3 nm when the value of RH decreased from 90% to 60%. Clearly, the slight wear of silicon at 90% RH was not caused by the wear of SiO_2_ tip. On the other hand, since the same loading parameters were used in all wear tests, the distinguishing wear of silicon at 60% RH and 90% RH should not be dominated by the mechanical interactions (see Section 4.1).

### 3.2. Evolution of the Wear Scar in KOH Solution

Even though the wear scar generated at 90% RH was located, it is still hard to characterize the damage of silicon by conventional equipment (TEM) [17,18,19]. Since the etching rate on the surface of SiO_x_ and Si(100) was significantly different in alkaline solution [20,21,22,23], the native oxide layer plays the role of etching mask for the substrate of Si(100) during the etching tests. Consequently, the Si(100) substrate would be quickly etched in alkaline solution if there was any damage on the native oxide layer. In order to investigate the damage of wearless scar on silicon sample surface at 90% RH, an indirect approach of etching experiment was introduced.

**Figure 3:**
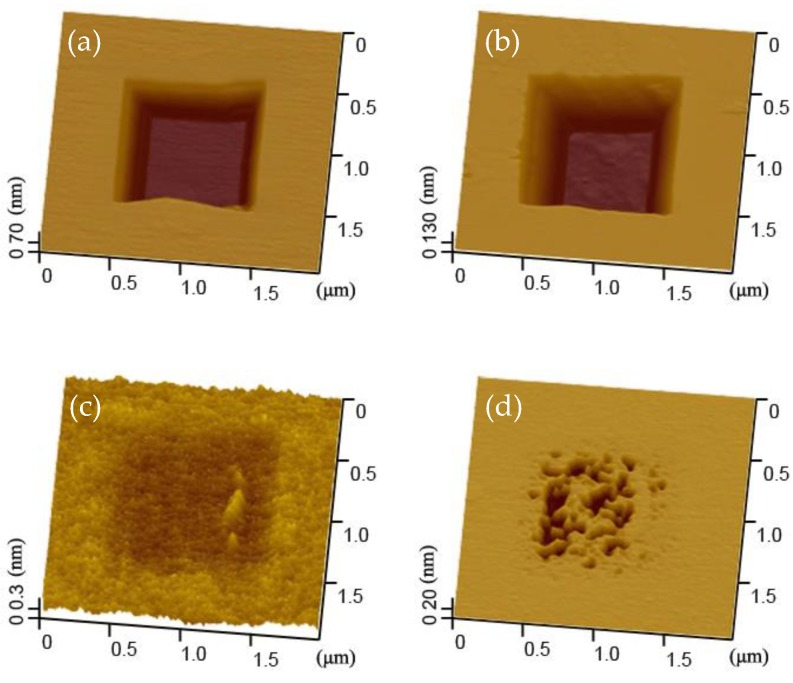
AFM images of wear scars on silicon surface after different etching time at 60%RH and 90%RH. *F_n_* = 3 μN, *N* = 2, *A* = 1 μm × 1 μm, *v* = 4 μm/s, *ω*_KOH_ = 10%. (**a**) Etching for 8 min after the wear test at 60% RH; (**b**) Etching for 16 min after the wear test at 60% RH; (**c**) Etching for 8 min after the wear test at 90% RH; (**d**) Etching for 16 min after the wear test at 90% RH.

**Figure 4:**
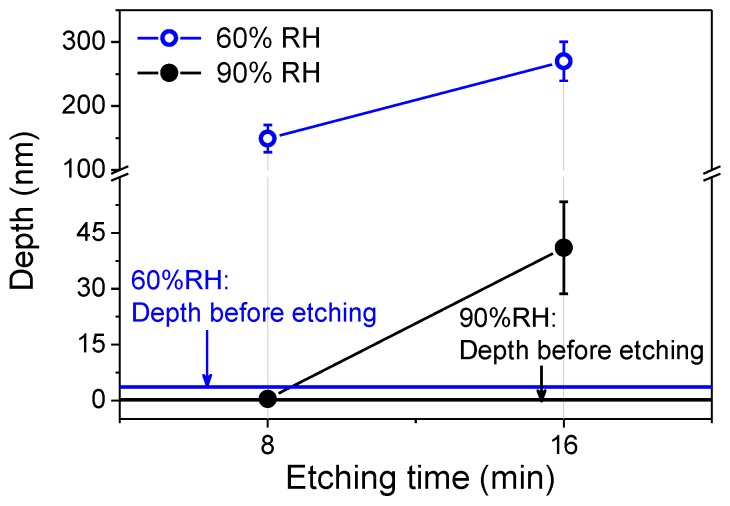
The depth of wear scars on silicon surface after different etching time between 60% RH and 90% RH. *F_n_* = 3 μN, *N* = 2, *A* = 1 μm × 1 μm, *v* = 4 μm/s, *ω*_KOH_ = 10%.

Figure 3 showed that the wear scar was quickly etched to a square pit after 8 min in alkaline solution for the case of 60% RH. Meanwhile, the depth of the wear scar increased from ~3 nm to ~150 nm, as shown in Figure 4. Given that the thickness of the native oxide layer (SiO_x_) on silicon sample surface was only 0.5 nm, the mask of SiO_x_ in the wear region was totally removed after the wear test at 60% RH. As a result, the etching pit would easily generate in the wear region without the etching mask of SiO_x_ [20,21,22,23]. Furthermore, the etching tests were also performed at 16 min to verify the etching resistance of the native oxide layer outside the wear region, as shown in Figure 3 and Figure 4. The present results implied that the wear scar could be further etched and the etching depth would increase to ~270 nm after 16 min. The results also demonstrated that there was no noticeable damage on the sample surface outside the wear region. Obviously, the native oxide layer in case of no damage could play a role of etching mask for at least 16 min under the given conditions. On the other hand, the etching tests for the case of 90% RH were also carried out, as shown in Figure 3 and Figure 4. It was found that a shallow square pit generated on the silicon sample surface after the etching test for 8 min. The depth of the wear scar increased to ~0.4 nm after the etching tests, as shown in Figure 4. Based on the above analysis, although the native oxide layer in the wear region was not totally removed after the wear tests at 90% RH, there was not zero damage to the silicon sample surface. According to the follow-up tests, the shallow pit would transform into a porous pit after 16 min. The deepest depth of this porous pit was around 41 nm, as shown in Figure 4. Therefore, there was no doubt that the silicon sample surface (covered by the native oxide layer) would be partly damaged after the wear tests at 90% RH. 

## 4. Discussion

### 4.1. Effect of Adhesion and Friction Force on the Wear Behaviors of Si/SiO_2_ Pair

**Figure 5:**
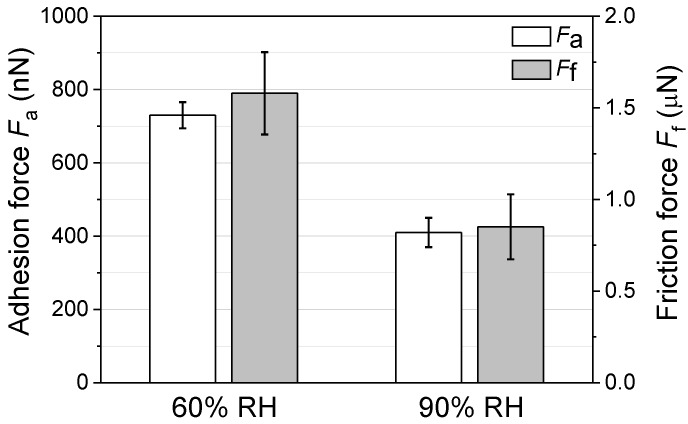
Adhesion force *F_a_* and friction force *F_f_* on silicon sample surface between 60% RH and 90% RH. *F_n_* = 3 μN, *N* = 2, *A* = 1 μm × 1 μm, *v* = 4 μm/s.

It is well known that the wear behaviors in micro/nano scale are affected by contact pressure [14,15]. Although the loading condition remained unchanged during the wear tests at 60% RH and 90% RH, the contact pressure for the pair of Si/SiO_2_ might still change with the variation of humidity [7,9,11]. Based on the contact theory established by Derjaguin, Muller and Toporov (DMT) [8,14,15,16], the contact pressure *P_c_* in the present study can be estimated by Equation (1).

(1) $$P_{c}=\frac{3}{2\pi }{\left[\frac{K^{2}}{R^{2}}\left(F_{n}+F_{a}\right)\right]}^{\frac{1}{3}}$$

where the combined elastic modulus *K* for the pair of Si/SiO_2_ is 64.8 GPa, the radius *R* of SiO_2_ tip is 1 μm, the applied normal load *F_n_* is set as 3 μN, and the adhesion force *F_a_* is tested in Figure 5.

Figure 5 showed that the adhesion force at 60% RH and 90% RH was around 730 nN and 410 nN, respectively. Thus, based on Equation (1), the contact pressure could be calculated as 1.19 GPa at 60% RH and 1.16 GPa at 90% RH. Since the contact pressure (< 1.2 GPa) in the present study is much smaller than the critical yield stress of silicon (7.0 GPa) [24,25], the contact of Si/SiO_2_ pair during the wear process will be elastic and the wear of silicon should not be dominated by the mechanical interactions, as shown in Figure 2.

The results mentioned above suggest that the wear of silicon may be dominated by tribochemical reactions (see Section 4.2). However, the activation energy of the tribochemical reactions is required to ensure the occurrence of these reactions [26,27]. Since the silicon sample is covered by the native oxide layer (SiO_x_), the key factor for the different wear of silicon samples is whether there is enough activation energy for the tribochemical wear of SiO_x_ in the wear region. In the present study, the activation energy of the native oxide layer (SiO_x_) refers to the bond energy of Si-O [7,11,28]. Therefore, the required energy *E_req_* to remove all the SiO_x_ in the wear region should be calculated to verify the existence of the native oxide layer after the wear tests. Here, the required energy *E_req_* to dissociate the chemical bonds of Si-O in the wear region is calculated as 3.5 × 10^−11^ J with Equation (2).

(2) $$E_{req}=n_{Si-O}E_{Si-O}\frac{V_{scar}}{V_{SiO}}$$

where the number of Si-O bonds *n_Si_*_-*O*_ in a silica molecule is 4, the bond energy *E_Si_*_-*O*_ of Si-O is 460 KJ/mol [29,30], the volume *V_scar_* of native oxide layer in the wear region is 5 × 10^−22^ m^3^, the molar volume *V_SiO_* of native oxide layer is 2.65 × 10^−5^ m^3^/mol [31,32].

Clearly, the native oxide layer on silicon sample surface will be totally removed only if the input energy during the wear process is higher than 3.5 × 10^−11^ J. Meanwhile, the wear of silicon sample will be slight if the input energy is lower than 3.5 × 10^−11^ J. In order to understand the different wear of silicon sample at 60% RH and 90% RH, the input energy during the wear process is calculated. The input energy generated in the wear tests, known as dissipated energy *E_dis_*, can be estimated by Equation (3) [33,34,35]. According to Equation (3), the dissipated energy *E_dis_* generated during the wear tests is estimated as 4.4 × 10^−11^ J and 2.4 × 10^−11^ J at 60% RH and 90% RH, respectively.

(3) $$E_{dis}=NF_{f}L\frac{W}{D_{con}}$$

where the width *W* of the wear region in the present study is 1 μm, the length *L* of wear region is 1 μm, the diameter *D_con_* of contact area is calculated as 71.4 nm [8,14,15,16], the friction force *F_f_* is tested in Figure 5, the number *N* of wear cycles is 2.

Given that the dissipated energy *E_dis_* at 60% is higher than the required energy *E_req_*, the native oxide layer on silicon sample surface will be totally removed by the tribochemical reactions. As a result, the wear of silicon sample at 60% RH was serious, as shown in Figure 2. On the other hand, since the dissipated energy *E_dis_* at 90% RH is lower than the required energy *E_req_*, the wear on the silicon sample is slight and the depth of the wear scar is less than 0.5 nm (thickness of the native oxide layer). It is obvious that the wear of silicon samples is still affected by mechanical interactions (including the friction force).

### 4.2. Effect of Water Structure on the Wear Mechanism of Si/SiO_2_ Pair

**Figure 6:**
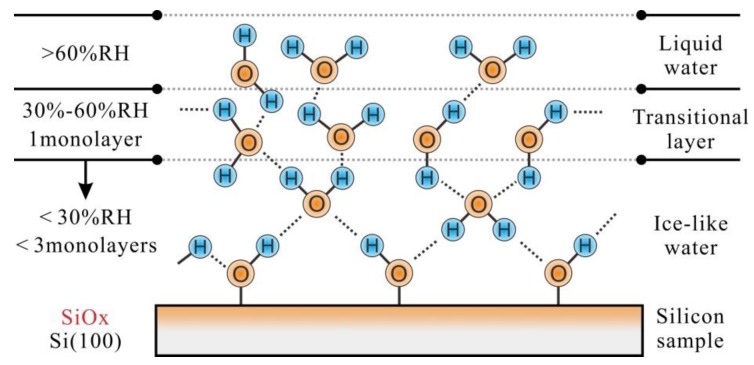
Water structure formed on silicon sample surface at various RHs.

Based on the experimental results, the absorbed water will form different structures on the silicon sample surface (covered by SiO_x_) in varied humid conditions [36,37]. At RH < 30%, the ice-like water with less than 3 monolayers will form on the SiO_x_ surface, see Figure 6. After that, the growth of the absorbed water is slow and less than one monolayer water forms on the surface of the ice-like water at 30–60% RH. With the further increase of RH value, the liquid water will generate on the previous water surface, as shown in Figure 6.

**Figure 7:**
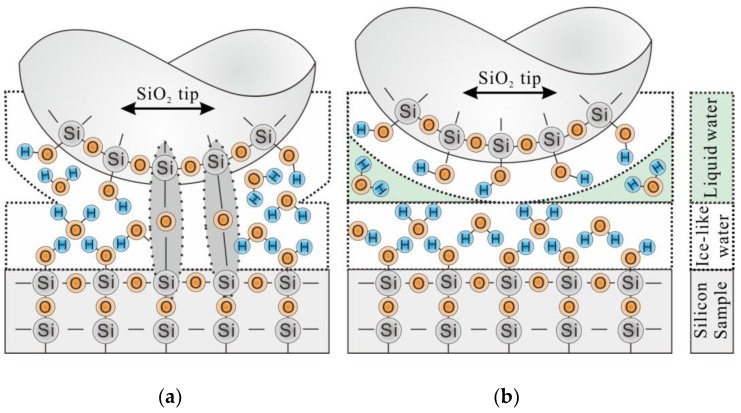
The illustration showing the wear mechanism of Si/SiO_2_ pair at (**a**) 60% RH and (**b**) 90% RH.

It is obvious that the contact between the interfaces of Si/SiO_2_ pair was different at 60% RH and 90% RH. At 60% RH, the interfaces of Si/SiO_2_ pair are separated by the ice-like water, as shown in Figure 7. For this condition, the Si-O-Si bridges will easily generate between the interfaces of Si/SiO_2_ pair, see Equation (4) [38].

≡Si-OH + HO-Si≡ → ≡Si-O-Si≡+H_2_O (4)

With the help of shearing action, the native oxide layer will be gradually removed by the Si-O-Si bridges [7,11]. After that, hydrolysis reactions will take place on the surface of Si(100) substrate, see Equation (5) [7,11,38].

≡Si-Si≡ +H_2_O → ≡Si-OH + H-Si≡ (5)

As a result, the surface of Si(100) substrate is terminated by the hydroxy, and the tribochemical Equation (4) can be maintained. Since the native oxide layer was removed by the Si-O-Si bridges at 60% RH, the dissipated energy *E_dis_* would not be lower than the required energy *E_req_*, see Section 4.1. However, the liquid water will form between the interfaces of Si/SiO_2_ pair at 90% RH. Due to the fluidity of the liquid water, the Si-O-Si bridges are hard to form between the interfaces of Si/SiO_2_ pair [28,36,38], as shown in Figure 7. For this reason, the shearing action (friction force) during the wear process has not to conquer the bond energy of Si-O-Si in the native oxide layer. Finally, the dissipated energy *E_dis_* is lower than the required energy *E_req_* and the wear of silicon sample was slight at 90% RH, see Section 4.1 and Figure 2.

If the wear mechanism proposed is right, the Si(100) substrate under the wear scar surface should be unwounded at 60% RH. In order to verify the wear mechanism, TEM tests were carried out in our previous research (for details see Reference [7]). The results suggested that the lattice of Si(100) substrate under the wear scar surface was not even changed. Moreover, the chemical reactions mentioned above were also proved in recent research using first-principles molecular dynamics (FPMD) methods [28]. It was clear that the serious wear on the silicon sample at 60% RH was caused by the intense tribochemical reactions. Meanwhile, the slight wear on the silicon sample at 90% RH was induced by the restrained tribochemical reactions. Even so, the surface of the silicon sample was still partly damaged at high humidity, as shown in Figure 3.

## 5. Conclusions

In this study, the wear of silicon sample (covered by the native oxide layer) was investigated at 60% RH and 90% RH with SiO_2_ spherical AFM tip. The wear on silicon samples at 60% RH and 90% RH was characterized by etching tests. The main conclusions are listed as following:

1. The wear of silicon sample was serious and very slight at 60% RH and 90% RH, respectively. Under the given conditions, the wear depth on silicon sample surface was around 3 nm at 60% RH. However, the wear scar was indistinct and the wear depth was close to the roughness of the silicon sample at 90% RH. Based on the repeated wear test at 60% RH, the slight wear at 90% RH was not caused by the wear of SiO_2_ tip;
2. At 60% RH, the native oxide layer was totally removed by the wear tests and the wear scar was etched to a deeper pit after the etching tests. On the other hand, after the etching tests for 16 min, a porous pit with the depth of ~41 nm would evolve from the indistinct wear scar at 90% RH;
3. Since the contact pressure (<1.2 GPa) in the present study was much smaller than the critical yield stress of silicon (7.0 GPa), the wear of silicon sample might be dominated by the tribochemistry instead of mechanical interactions. Even so, the tribochemical reactions would be still affected by the mechanical interactions in the form of the dissipated energy;
4. Further analysis indicated that the tribochemical reactions were related to the formation of Si-O-Si chemical bond bridges between the interfaces of Si/SiO_2_ pair. Due to the different water structures at various RHs, the formation of the chemical bond bridges would be facilitated and restrained at 60% RH and 90% RH, respectively. As a result, the dissipated energy and the wear of silicon sample were different at 60% RH and 90% RH.

These results suggest that the wear resistance of silicon-based MEMS working in humid conditions could be improved by increasing the RH value. Meanwhile, polishing quality on silicon surfaces could be improved using the tribochemical reactions during the chemical–mechanical polishing (CMP) process [39]. Moreover, since the adhesion force was different between 60% RH and 90% RH, the capture and release process of silicon-based microgrippers could be achieved by changing the RH value [40].
